# The Significance of CEA and CA 19-9 Levels in Serum and Peritoneal Fluid in Colorectal Cancer Patients in the Context of Peritoneal Metastases and Cytology Results

**DOI:** 10.3390/cancers17162661

**Published:** 2025-08-15

**Authors:** Michał Bąk, Magdalena Wojciech, Roman Monczak, Marek Zawadzki, Dawid Murawa

**Affiliations:** 1 General and Oncological Surgery, The Karol Marcinkowski University Hospital, 65-417 Zielona Góra, Poland; 2 Department of Surgery and Oncology, University of Zielona Góra, 65-417 Zielona Góra, Poland; 3 Department of Applied Mathematics, Institute of Mathematics, University of Zielona Góra, 65-417 Zielona Góra, Poland; 4 Department of Pathology, The Karol Marcinkowski University Hospital, 65-417 Zielona Góra, Poland; 5 Subdepartment of Metabolic Surgery and the Subdepartment of Endocrine Surgery, Department of General and Minimally Invasive Surgery, Provincial Specialist Hospital in Wrocław Research and Development Center, 51-124 Wrocław, Poland; marek.zawadzki@pwr.edu.pl; 6 Department of Clinical Surgical Sciences, Faculty of Medicine, Wrocław University of Science and Technology, 50-368 Wrocław, Poland; 7 Provincial Hospital in Poznań, 60-479 Poznań, Poland

**Keywords:** CA 19-9, carcinoembryonic antigen (CEA), colon cancer, peritoneal carcinomatosis, peritoneal washings, tumor markers, statistical tests, optimization methods

## Abstract

Background/Objectives: Colorectal cancer (CRC) frequently metastasizes to the peritoneum, significantly worsening patient prognosis. While serum tumor markers such as carcinoembryonic antigen (CEA) and carbohydrate antigen 19-9 (CA 19-9) are routinely measured, their diagnostic or prognostic role in peritoneal fluid remains unclear. This study aimed to assess the relationship between CEA and CA 19-9 levels in both serum and peritoneal fluid, and the clinical stage of CRC, particularly focusing on the presence of peritoneal metastases and positive cytology. Methods: We retrospectively analyzed data from 89 patients with histologically confirmed CRC who underwent surgery between 2020 and 2023. All patients had preoperative assessment of CEA and CA 19-9 levels in serum and peritoneal fluid, along with cytological examination of peritoneal fluid samples. Patients were categorized based on the presence or absence of macroscopic peritoneal metastases and cytology results. Results: Elevated levels of CEA and CA 19-9 in peritoneal fluid were significantly associated with the presence of peritoneal metastases. A positive cytological finding also correlated with higher marker concentrations. Conclusions: CEA and CA 19-9 levels in peritoneal fluid strongly correlate with peritoneal dissemination in CRC. These markers may serve as additional predictive factors, aiding in early detection of peritoneal spread and improved risk stratification. Their assessment may be useful in guiding intraoperative and postoperative decision-making.

## Simple Summary

Colorectal cancer (CRC) often metastasizes to the peritoneum, significantly worsening the prognosis. Although tumor markers such as CEA and CA 19-9 are routinely determined in serum, their significance in peritoneal fluid remains poorly understood. This study aimed to evaluate the relationship between serum and peritoneal fluid levels of CEA and CA 19-9 and the stage of colorectal cancer (including the presence of peritoneal metastases and the presence of tumor cells on cytologic examination).

## 1. Introduction

Colorectal cancer ranks among the most frequently diagnosed cancers worldwide. In 2022, an estimated 1.9 million new cases of colorectal cancer occurred globally, representing 9.6% of all new cancer cases. Colorectal cancer is the second-leading cause of cancer death, with approximately 900,000 deaths (9.3% of all cancer deaths), according to data from the International Agency for Research on Cancer (IARC), as reported by Bray et al. [1].

In Europe, colorectal cancer is among the most common cancers, ranking second in women and third in men. It accounts for more than 12% of all cancer-related deaths in Europe [2]. Peritoneal metastasis is one of the main paths of colorectal cancer recurrence after surgery and a common cause of death among patients with this cancer [3]. Although the diagnostic and therapeutic management of colorectal metastasis to the liver is widely discussed with standardized treatment methods in place, local recurrence and intraperitoneal spread remain relatively less investigated. Likewise, the lack of well-established therapeutic approaches leads to a poorer prognosis.

Peritoneal metastasis represents a major challenge in the management of resectable colorectal cancer, as it affects more than 10% of patients who have not had any macroscopic peritoneal lesions intraoperatively [4].

Currently, the diagnosis of tumor cells in the peritoneum is performed through cytologic examination of peritoneal fluid, which helps detect microscopic metastases invisible to the naked eye.

Positive peritoneal fluid cytology results have been associated with a less favorable prognosis for gynecological cancers as well as gastric cancer [5,6,7,8]. However, their role as a prognostic factor in colorectal cancer remains elusive [9,10,11,12].

Although cytologic examination of peritoneal washings is considered the standard for detecting free tumor cells in the peritoneal cavity, its sensitivity in colorectal cancer is inadequate, ranging from 2.2% to 51.7%, depending on the tumor stage and method used [13].

There is a need to develop new biomarkers that will increase the efficiency of diagnosing peritoneal metastases. Recent studies, including a previous study by the authors of this paper [14], indicate that tumor markers detected in peritoneal fluid or lavage in patients with gastric cancer show high sensitivity and specificity in detecting peritoneal spread [14,15].

In recent years, increasing attention has been given to the role of circulating tumor DNA (ctDNA) in detecting minimal residual disease (MRD) and metastatic spread. However, studies by Bando et al. [16] and Crisafulli et al. [17] have shown that peritoneal and pulmonary metastases in colorectal cancer are associated with very low levels of ctDNA in peripheral blood. This limitation reduces the sensitivity of ctDNA-based MRD detection in such patients. Therefore, measuring tumor markers such as CEA and CA 19-9—both in serum and in peritoneal fluid—may offer valuable complementary biomarkers for assessing the tumor burden, especially in cases where molecular detection falls short.

The purpose of the present study is to evaluate the relationship between the levels of tumor markers, such as carcinogen-embryonic antigen (CEA) and CA 19-9 antigen, determined in blood serum (sCEA and sCA 19-9) and in peritoneal fluid obtained during surgery (pCEA and pCA 19-9), and the stage of colorectal cancer, including the presence of tumor cells in peritoneal fluid cytology.

## 2. Materials and Methods

### 2.1. General Information

Eighty-nine patients with histopathologically confirmed colon cancer, surgically treated at the Single Surgical Department, were enrolled in this prospective study from May 2021 to May 2024.

### 2.2. Research Methods

Preoperative serum samples were collected from all patients for tumor marker detection, including CEA and CA 19-9. Immediately after opening the abdominal wall or the establishment of pneumoperitoneum, 200 mL of 0.9% NaCl solution, preheated to 37 °C, was pumped into the peritoneal cavity, primarily in the area of the tumor. The fluid was then manually distributed throughout the cavity. From the lavage, 100 mL was collected into a sterile container for cytological examination, and 5 mL was used to determine CEA and CA 19-9 concentrations. In this cohort, no free peritoneal fluid was observed intraoperatively; therefore, peritoneal lavage was performed in all cases using the described method. The material for cytological analysis was then promptly examined (delivered within 15 min to the Department of Pathomorphology for examination), while the portion of the fluid used for tumor marker measurement was analyzed on an ad hoc basis.

The samples collected for cytological examination were centrifuged using the Cytospin method for 3 min at 1000 rpm. The precipitate was then evenly spread onto slides, fixed with Cytofix, and subsequently stained with the H-E method (hematoxylin and eosin) (DAKO, Acquired by Agilent Technologies (US-based) in 2012 (Santa Clara, CA, USA)). H-E staining is used for routine examinations. If any suspicious findings, such as atypical cells, were detected, additional immunohistochemical testing was performed. The cytological examination result was considered positive in the presence of viable cancer cells.

CEA and CA 19-9 levels were measured using a chemiluminescent enzyme-linked immunosorbent assay (Roche, Basel, Switzerland). Serum tumor marker levels were also measured using this method before surgery.

**Figure 1:**
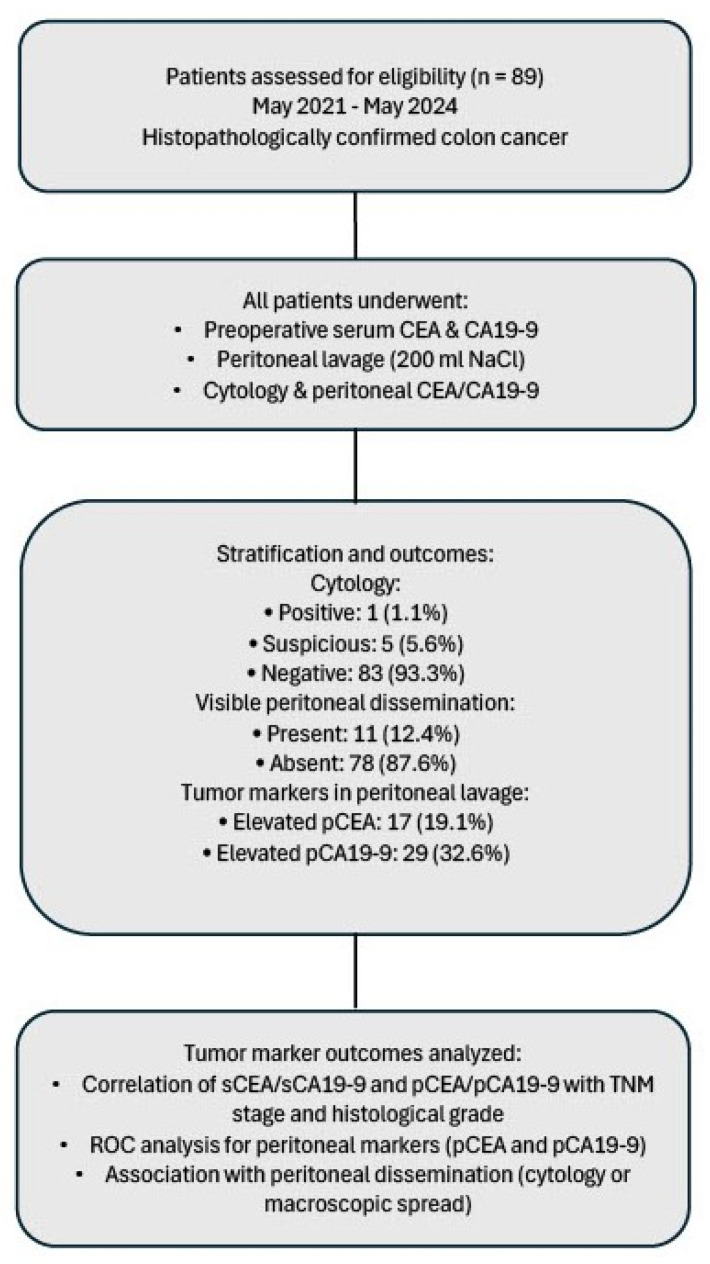
Flow diagram of patient inclusion, procedures performed, stratification by cytology and peritoneal carcinomatosis, and tumor marker analysis.

The study design and patient stratification are summarized in Figure 1.

### 2.3. Statistical Analysis

Statistical analysis was performed using the statistical software package R (R version 4.3.2) [18], which is distributed under an open-source license. Continuous variables were reported as means ± standard deviations and medians ± lower and upper quartiles. Categorical variables were expressed as frequencies and percentages. The distribution of quantitative variables was tested with the Shapiro–Wilk test. Under a normal distribution, the equality of variance of the variables in the comparable groups was tested using the Bartlett test. The statistical significance of intergroup differences was compared using Student’s t-test, Welch’s test, or the non-parametric Wilcoxon rank–sum test for continuous variables. The χ2 test or Fisher’s exact test was used to compare categorical variables. Fisher’s exact test of independence was used when the assumptions of the chi-square test were not held. Additionally, Spearman’s rank correlation was used to assess the relationships between markers. The level of statistical significance was defined as 0.05 for all statistical tests.

Patients were divided into different groups according to positive or negative cytology results as well as the presence or absence of visible intraperitoneal dissemination. Patient demographics and tumor characteristics, including the differentiation grade, T and N stages, and vascular, lymphatic, and neuronal invasion, were analyzed. The results of morphology and kidney function (based on creatinine and GFR levels) were also included in the analysis. Patients were classified based on the sCEA and sCA 19-9 levels into groups with low (≤3 ng/mL and ≤37 µ/mL) or high (>3 ng/mL and >37 µ/mL) values of these markers.

ROC curve analysis and AUC were used to determine the optimal cut-off values for the levels of peritoneal tumor markers pCEA and pCA 19-9 to predict the development of peritoneal tumor spread [19]. In our study, the Youden index was used for this purpose, which allowed us to identify threshold values that maximize both sensitivity and specificity. The bootstrap method was used to estimate the *p*-value for the Youden index, generating 1000 resamples [20].

The definition of tumor dissemination into the peritoneum included a positive cytology result during the surgery or visible intraperitoneal dissemination. Patients were divided into groups with low or high levels of peritoneal tumor markers according to the cut-off values to assess their impact on the development of tumor dissemination into the peritoneum.

This study was approved by the Bioethics Committee of the District Medical Chamber in Zielona Góra under resolution number 10/146/2021 and followed the principles of the Declaration of Helsinki. All patients provided written informed consent to participate in this study.

## 3. Results

### 3.1. Features of the Patient Cohort

The study included 89 patients, with a mean age of 69 years. In this group, 46 (51.7%) patients were men and 43 were women (48.3%). Among them, 16 (18%) patients received neoadjuvant chemotherapy, and 20 (22.5%) received preoperative radiation therapy. In five cases, the cytology results raised suspicion of intraperitoneal spread, which warranted additional immunohistochemical diagnosis. A positive cytology result was reported in 1 patient (1.1%), while the presence of intraperitoneal spread was found in 11 patients (12.4%). Distant metastases were present in 22 (24.7%) patients.

### 3.2. Serum and Peritoneal Tumor Markers

#### 3.2.1. Serum and Peritoneal Marker Levels

Standards for serum levels of tumor markers were adopted according to local laboratory guidelines: sCEA (≤3 ng/mL) and sCA 19-9 (≤37 µ/mL). Elevated serum levels of tumor markers were observed in 53 (59.6%) patients for the CEA marker 43 (48.3%) and for CA 19-9 in 17 (19.1%) patients, of which 15 patients had both sCEA and sCA 19-9 elevated at the same time.

#### 3.2.2. Diagnostic Performance of Peritoneal Markers

**Figure 2:**
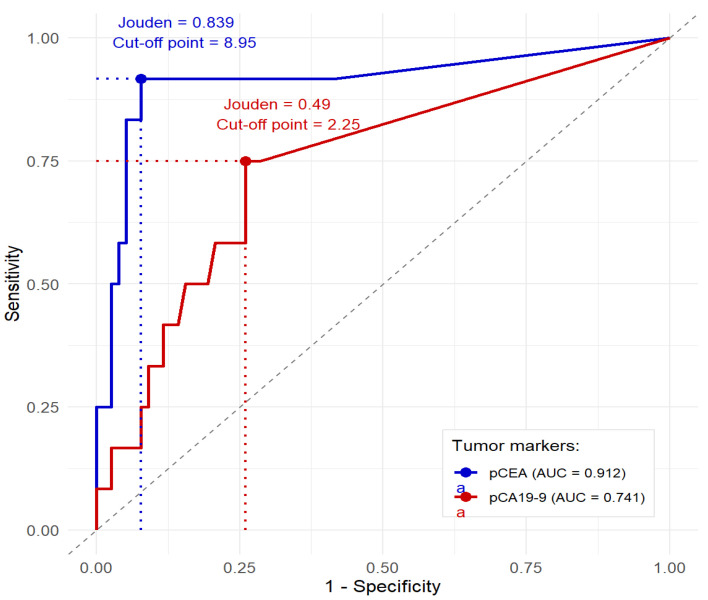
Receiver operating characteristic (ROC) curves for the markers pCEA and pCA 19-9 measured in peritoneal fluid. Points on the curves indicate optimal values that maximize both sensitivity and specificity. The description in the legend also includes the value of the area under the ROC curve. For each marker, the cut-off value and the Youden statistic value are also provided.

ROC curve analysis was performed to determine threshold values for peritoneal markers (pCEA and pCA 19-9) (Figure 2). Considering sensitivity and specificity, these values were determined based on the diagnosis of peritoneal spread during surgery or by positive cytology results. The analysis showed that the optimal threshold values for pCEA and pCA 19-9 are 8.95 ng/mL and 2.25 µ/mL, respectively. The corresponding sensitivity and specificity values are shown in Table 1.

Both pCEA and pCA 19-9 markers demonstrated highly significant discriminatory ability (respectively, *p*-value = 0.002 and *p* < 0.001). Of the indicators analyzed, pCEA had the highest AUC, achieving a sensitivity of 91.7% and specificity of 92.2% at the established threshold. The pCA 19-9 indicator showed lower sensitivity and specificity of 75% and 74%, respectively.

Based on the analysis, elevated levels of tumor markers in abdominal washings were found in 17 patients (19.1%) for CEA and in 29 patients (32.6%) for CA 19-9.

#### 3.2.3. Association with TNM Parameters and Other Features

There was no significant association between levels of tumor markers in serum and peritoneal lavage and demographic data, history of neoadjuvant chemotherapy, or positive peritoneal cytology. There was also no correlation with blood count or renal function (based on creatinine level or GFR); only the sCEA level correlated statistically significantly with the patient’s Hgb and HCT levels and prior neoadjuvant radiotherapy (*p* < 0.05).

A correlation was observed between sCEA and pCEA levels and tumor stage (T, N, M, V, L, Pn) or histological tumor grade (*p* < 0.05). Notably, the association with sCA 19-9 and pCA 19-9 was limited to tumor stage, and not its histological grade (*p* > 0.05) (Table 2).

#### 3.2.4. Inter-Marker Correlation

Table 3 shows the Spearman test correlation coefficients for the association between numerical marker values. At a significance level of 0.05, a statistically significant positive monotonic correlation was found for each pair of markers. The strongest correlation was observed between the CEA marker pair and between the CA 19-9 markers.

### 3.3. Factors Affecting the Development of Peritoneal Carcinomatosis

In the study group, peritoneal carcinomatosis was found in 11 patients (12.4%). The median age of patients without peritoneal carcinomatosis was 68 years, and among those with peritoneal carcinomatosis, the median age was 69 years. A statistically significant correlation was found between the presence of peritoneal carcinomatosis and the presence of distant metastases (*p* < 0.001). The patient who showed a positive cytology result did not belong to the group of patients with peritoneal carcinomatosis, and thus statistical significance could not be observed.

In patients with peritoneal carcinomatosis, elevated values of pCEA, sCA 19-9, and pCA 19-9 were observed (*p* < 0.05), but such a relationship was not observed for sCEA (*p* > 0.05).

The analysis showed a statistically significant relationship between the presence of peritoneal carcinomatosis and tumor grade on the TNM scale (to each of the individual components), as well as the histological grade (Table 4).

### 3.4. Relationship Between Peritoneal Cytology Results and Clinicopathological Factors

This study showed the presence of free tumor cells in the peritoneal fluid in 1 patient out of 89 tested.

A statistical comparison of patients with positive and negative cytology results for tumor cells yielded no significant differences between the two groups in terms of gender, age, distant metastasis or intraperitoneal spread, history of neoadjuvant therapy, and levels of CEA and CA 19-9 markers in both serum and peritoneal lavage. The location, necrosis, and mucinous nature of the tumor; grade; morphology; and renal function (based on creatinine or GFR levels) also presented no statistical relationship (*p* > 0.05).

The patient with a positive cytology result did not show symptoms of macroscopic intraperitoneal spread.

The limited number of cases with positive cytology makes it impossible to treat these results as statistically significant.

## 4. Discussion

The findings of this study indicate that measuring tumor markers CEA and CA 19-9 in peritoneal lavage fluid (pCEA and pCA 19-9) may serve as a valuable tool in assessing the advancement of colorectal cancer (CRC), particularly in the context of peritoneal dissemination. Statistically significant correlations were observed between elevated levels of these markers and both peritoneal metastases and a higher disease stage according to the TNM classification. Notably, this association was present even in cases without positive cytology or visible macroscopic dissemination.

Cancer cells can spontaneously emerge from the serosal surface of the intestine, with or without damage to the serosal membrane, and can spread within the peritoneal cavity. Hase et al. [21] described seven factors associated with positive cancer cytology.

According to the study, positive peritoneal cytology is an independent risk factor for reduced cancer-specific survival, as well as lower rates of peritoneal recurrence-free survival [9,10,21,22,23,24].

Bosanquet et al. [25] conducted a meta-analysis in 2013 that evaluated the impact of positive abdominal washout cytology on the risk of local, distant, and overall recurrence, as well as survival rates. The results showed that cytologically positive peritoneal lavage remained a significant predictor of increased mortality, and the risk of recurrence was significantly higher in the group with cytologically positive lavage.

As of now, accurate detection of small cancer metastases in the peritoneal cavity of patients with colorectal cancer remains challenging.

Traditional cytological examination remains an essential tool in diagnosis, enabling the identification of free-floating cancer cells. However, standard cytologic methods can have limitations that reduce their effectiveness.

Bosanquet et al. [25], in a meta-analysis, estimated the mean detection rates (yield rates) of cancer cells in peritoneal fluid from colorectal cancer patients to be 8.4% for conventional cytology, 28.3% for immunocytology (ICC), and 14.5% for PCR. In this meta-analysis, the percentage of detected free tumor cells in the peritoneal cavity ranged from 2.2% to 41%, with a weighted average of 11.6%.

The small number of tumor cells released into the peritoneal cavity in the early stages of the disease, and their similarity to reactive mesothelial or inflammatory cells present in exudative fluids make them difficult to detect by cytologic methods.

Cytological examination for colorectal cancer is used less frequently than in gastric or ovarian cancers. Due to the difficulty in differentiating colorectal adenocarcinomas from reactive and inflammatory lesions, its diagnostic value in evaluating typical lesions in this organ is limited [26].

For this reason, peritoneal fluid cytology is not routinely performed in patients with colorectal cancer, and the fluid itself is not considered a reliable clinical indicator. To compensate for the low sensitivity of cytology, many authors have tried to diagnose tumor effusion by analyzing tumor markers. Biochemical markers can significantly enhance the sensitivity of peritoneal fluid analysis, although cytology remains a crucial prognostic factor.

Due to the limited effectiveness of molecular methods based on ctDNA in detecting peritoneal dissemination—as documented by Bando et al. [16] and Crisafulli et al. [17]—tumor markers present in peritoneal fluid may serve as a valuable complement to existing diagnostic strategies in patients with colorectal cancer. These studies demonstrated that patients with metastases confined to the lungs or peritoneum exhibit significantly lower levels of ctDNA, which directly impacts the sensitivity of detecting subclonal genetic variants. In such cases, relying solely on plasma-based analysis may lead to false-negative results.

Although ctDNA analysis was not included in this study, our results demonstrate that pCEA and pCA 19-9 levels correlate with clinical and pathological features of disease advancement, suggesting their potential role in risk stratification and therapeutic decision-making.

Many studies indicate that tumor markers CEA and CA 19-9 collected from the peritoneal cavity can be a reliable indicator of peritoneal metastasis and prognosis in patients with gastric cancer [14,27,28], and, according to a study by Li JK et al., determination of pCEA compared to intraoperative cytology is a more sensitive and reliable indicator of peritoneal metastasis and prognosis in patients with gastric cancer [29].

In our study, tumor markers CEA and CA 19-9 in peritoneal fluid correlated with some clinicopathological factors, including intraperitoneal spread. Moreover, no such correlation was shown by cytological examination. However, all samples analyzed in this study were derived from peritoneal lavage, not from natural (free) peritoneal fluid. Therefore, comparisons with studies based on free peritoneal fluid should be interpreted cautiously due to potential dilution effects and differences in sampling methods.

In a study by YunChang Tan et al. [30], it was shown that the markers pCA 125, pCEA, and pCA 19-9 could potentially be used to differentiate colorectal malignancies. According to their study, colorectal cancer marker pCA 19-9 showed high sensitivity and specificity in detecting both macro- and micro-metastases in the peritoneum. In our study, pCEA manifested slightly better results than pCA 19-9, though both markers had high sensitivity and specificity.

In a study by Lee et al. [31], they showed that the level of CEA in peritoneal fluid was significantly correlated with peritoneal cytology and the tumors’ histologic types, whereas the level of CA 19-9 showed a significant correlation with peritoneal metastasis, depth of primary tumor infiltration, distant metastasis, stage of disease, peritoneal cytology, and histological type. There was no significant correlation between serum CA 19-9 levels and CA 19-9 levels in peritoneal fluid (*p* = 0.347). However, our study arrived at the opposite conclusion, as both pairs of markers showed a strong correlation.

Moreover, Lee et al. also assumed that pCEA and pCA 19-9 levels were associated with disease-free survival and overall survival. In patients with negative cytological results, CEA levels in peritoneal fluid above 4 ng/mL significantly correlated with recurrence and peritoneal metastasis.

Some studies have analyzed the relationship between tumor markers taken from blood serum and those obtained from peritoneal fluid. In a study by Fang Liu et al. [32], tumor markers in peritoneal fluid showed higher diagnostic sensitivity than those determined in serum. In contrast, combining cytology with markers in peritoneal fluid increased sensitivity to 94%, with 97% specificity. These results confirm that the use of tumor markers in tandem with cytology significantly improves the diagnosis of malignant ascites. Therefore, measurement of CEA and CA 19-9 in peritoneal fluid can complement cytology.

Further supporting this connection is the study by Fang Liu et al., in which analysis of marker levels in serum and peritoneal fluid showed that in patients with elevated serum markers, high levels of the same markers could be detected in peritoneal fluid. However, some patients whose peritoneal fluid tested positive did not have elevated serum marker values, which would suggest that peritoneal fluid marker analysis is more diagnostically valuable than serum.

Our purpose was to evaluate the relationship between tumor marker levels and the stage of colorectal cancer, to enable earlier detection of peritoneal metastases. The results of this study highlight important clinical applications that may help, for example, in detecting peritoneal metastases before surgery, which would prove a worthwhile supplement, or even alternative, to computer tomography (CT).

Previous studies have shown that the sensitivity of CT in detecting peritoneal metastases hinges on the sizes of the lesions—nodules smaller than 0.5 cm are detected by CT with only 11% sensitivity [33]. In such cases, performing abdominal lavage via peritoneal puncture and testing the fluid for tumor markers could help avoid unnecessary surgery.

The above may also prove insightful during surgery when no macroscopic metastases are found during intraoperative exploration. In such cases, tumor markers in the lavage fluid could detect the presence of residual cancer cells. If they are present, then preventive intervention, such as intraperitoneal perfusion chemotherapy, could be performed to minimize the risk of early tumor recurrence after surgery.

## 5. Conclusions

This study has shown that the presence of tumor markers in the lavage fluid (pCEA and pCA 19-9) has a significant association with tumor progression. In addition, the level of these markers is a predictor of intraperitoneal spread. Detection of tumor markers in lavage could significantly complement cytology in the diagnosis of colorectal cancer staging. The presence of elevated levels of pCEA and pCA 19-9 in patients with colorectal cancer may have significant prognostic significance and could prove helpful in treatment planning.

The role of the aforementioned markers in serum and peritoneal fluid (especially in the diagnosis of cancer progression) should be evaluated in prospective studies.

## 6. Limitations

Unlike our previous study on gastric cancer [14], in which free fluid was found in the peritoneal cavity in some patients, in our study group, no such fluid was noted during surgery. Therefore, peritoneal lavage with saline solution was performed on all patients. This methodological difference should be taken into account when interpreting the comparability of tumor marker concentrations in peritoneal fluid between the two analyses.

## Figures and Tables

**Table 1:** Diagnostic characteristics of tumor markers pCEA and pCA 19-9 in peritoneal fluid: AUC, Youden index, optimal marker cut-off value, specificity, and sensitivity.

Marker	AUC	Youden Index	Youden Index *p*-Value	Cut-Off Value	Sensitivity (%)	Specificity (%)
pCEA	0.912	0.839	0.002	8.95	91.7	92.2
pCA 19-9	0.741	0.49	<0.001	2.25	75	74

**Table 2:** Factors associated with the tumor markers CEA and CA 19-9 (values in the table represent *p*-values from the corresponding statistical tests). Tumor location includes the following anatomical sites: cecum, ascending colon, transverse colon, descending colon, sigmoid colon, and the intraperitoneal part of the rectum. Abbreviations: T, N, M—components of the TNM classification (tumor depth, nodal involvement, distant metastasis), L—lymphatic invasion, V—vascular invasion, Pn—perineural invasion (additional prognostic factors reported alongside TNM).

Variable	CEA		CA 19-9	
	Serum	Peritoneal	Serum	Peritoneal
Cytology	0.483	0.191	0.193	0.326
Neoadjuvant radiotherapy	0.002	0.34	1	1
Neoadjuvant chemotherapy	0.074	0.727	0.501	0.449
Mucinous characteristic component	0.341	0.322	1	0.721
Tumor necrosis	1	1	0.327	1
Histopathological stage	0.022	0.002	0.689	0.668
Tumor location	0.065	0.246	0.274	0.655
T	0.002	<0.001	0.026	0.015
N	0.014	<0.001	<0.001	0.028
M	<0.001	<0.001	<0.001	<0.001
L	<0.001	<0.001	0.005	0.024
V	0.037	<0.001	0.018	0.029
Pn	<0.001	<0.001	0.002	0.021
WBC	0.559	0.108	0.681	0.123
HGB	0.035	0.426	0.597	0.667
HCT	0.041	0.057	0.184	0.107
PLT	0.092	0.872	0.389	0.883
Creatinine	0.849	1	1	0.505
GFR	0.761	0.874	0.516	0.255

**Table 3:** Spearman rank correlation values and *p*-values of the correlation test between serum and peritoneal marker levels.

Marker	CA 19-9 in Abdominal Cavity	CA 19-9 in Serum	CEA in Abdominal Cavity	CEA in Serum
CA 19-9 in abdominal cavity	-			
CA 19-9 in serum	0.612 (<0.001)	-		
CEA in abdominal cavity	0.398 (<0.001)	0.394 (<0.001)	-	
CEA in serum	0.321 (0.002)	0.371 (<0.001)	0.653 (<0.001)	-

**Table 4:** Statistical relationship between the variables and peritoneal dissemination.

Clinicopathologic Factors	Peritoneal Carcinomatosis		*p*-Value
	Negative, n (%)	Positive, n (%)	
Sex			0.905
Female	37 (47.4%)	6 (54.5%)	
Male	41 (52.6%)	5 (45.5%)	
Cytology			1.00
Negative	77 (98.7%)	11 (100.0%)	
Positive	1 (1.3%)	0 (0.0%)	
Distant Metastases			0.024
Negative	62 (79.5%)	5 (45.5%)	
Positive	16 (20.5%)	6 (54.5%)	
CEA in Serum			0.159
Beyond the norm	35 (44.9%)	8 (72.7%)	
Norm	43 (55.1%)	3 (27.3%)	
CEA in Abdominal Cavity			<0.001
Beyond the norm	7 (9.0%)	10 (90.9%)	
Norm	71 (91.0%)	1 (9.1%)	
CA 19-9 in Serum			0.006
Beyond the norm	11 (14.3%)	6 (54.5%)	
Norm	66 (85.7%)	5 (45.5%)	
CA 19-9 in Abdominal Cavity			0.005
Beyond the norm	21 (26.9%)	8 (72.7%)	
Norm	57 (73.1%)	3 (27.3%)	
Neoadjuvant Radiotherapy			0.444
Negative	59 (75.6%)	10 (90.9%)	
Positive	19 (24.4%)	1 (9.1%)	
Neoadjuvant Chemotherapy			0.681
Negative	63 (80.8%)	10 (90.9%)	
Positive	15 (19.2%)	1 (9.1%)	
Mucinous Characteristic Component			0.223
Negative	64 (82.1%)	7 (63.6%)	
Positive	14 (17.9%)	4 (36.4%)	
Tumor Necrosis			0.558
Negative	73 (93.6%)	10 (90.9%)	
Positive	5 (6.4%)	1 (9.1%)	
Histopathological Stage			0.002
G1	52 (66.7%)	3 (27.3%)	
G2	15 (19.2%)	1 (9.1%)	
G3	11 (14.1%)	7 (63.6%)	
Tumor Location			0.085
Ascending colon	11 (14.1%)	2 (18.2%)	
Cecum	13 (16.7%)	4 (36.4%)	
Descending colon	2 (2.6%)	1 (9.1%)	
Intraperitoneal rectum	43 (55.1%)	2 (18.2%)	
Sigmoid colon	5 (6.4%)	1 (9.1%)	
Transverse colon	4 (5.1%)	1 (9.1%)	
T Stage			<0.001
0	1 (1.3%)	0 (0.0%)	
1	2 (2.6%)	0 (0.0%)	
2	21 (26.9%)	0 (0.0%)	
3	43 (55.1%)	1 (9.1%)	
4	11 (14.1%)	10 (90.9%)	
N Stage			<0.001
0	40 (51.3%)	0 (0.0%)	
1	23 (29.5%)	1 (9.1%)	
2	15 (19.2%)	10 (90.9%)	
M Stage			<0.001
0	63 (80.8%)	0 (0.0%)	
1	15 (19.2%)	11 (100.0%)	
L Stage			<0.001
0	59 (75.6%)	0 (0.0%)	
1	19 (24.4%)	11 (100.0%)	
V Stage			<0.001
0	63 (80.8%)	1 (9.1%)	
1	15 (19.2%)	10 (90.9%)	
Pn Stage			<0.001
0	61 (78.2%)	1 (9.1%)	
1	17 (21.8%)	10 (90.9%)	

## Data Availability

The data presented in this study are available on request from the corresponding author due to legal (internal hospital data, where the study took place) and ethical reasons.

## Abbreviations

The following abbreviations are used in this manuscript:

AUC	Area Under the Curve
CA	Carbohydrate Antigen (część CA 19-9)
CEA	Carcinoembryonic Antigen
CRC	Colorectal Cancer
GFR	Glomerular Filtration Rate
HCT	Hematocrit
HGB	Hemoglobin
PLT	Platelets
ROC	Receiver Operating Characteristic
WBC	White Blood Cells
